# Respiratory epithelial cells require Toll-like receptor 4 for induction of Human β-defensin 2 by Lipopolysaccharide

**DOI:** 10.1186/1465-9921-6-116

**Published:** 2005-10-12

**Authors:** Ruth MacRedmond, Catherine Greene, Clifford C Taggart, Noel McElvaney, Shane O'Neill

**Affiliations:** 1 Department of Respiratory Research, Royal College of Surgeons in Ireland, Beaumont Hospital, Dublin 9, Ireland

**Keywords:** Airway epithelium, Toll-like Receptor 4, Lipopolysaccharide, Human β-defensin 2.

## Abstract

**Background:**

The respiratory epithelium is a major portal of entry for pathogens and employs innate defense mechanisms to prevent colonization and infection. Induced expression of human β-defensin 2 (HBD2) represents a direct response by the epithelium to potential infection. Here we provide evidence for the critical role of Toll-like receptor 4 (TLR4) in lipopolysaccharide (LPS)-induced HBD2 expression by human A549 epithelial cells.

**Methods:**

Using RTPCR, fluorescence microscopy, ELISA and luciferase reporter gene assays we quantified interleukin-8, TLR4 and HBD2 expression in unstimulated or agonist-treated A549 and/or HEK293 cells. We also assessed the effect of over expressing wild type and/or mutant TLR4, MyD88 and/or Mal transgenes on LPS-induced HBD2 expression in these cells.

**Results:**

We demonstrate that A549 cells express TLR4 on their surface and respond directly to *Pseudomonas *LPS with increased HBD2 gene and protein expression. These effects are blocked by a TLR4 neutralizing antibody or functionally inactive TLR4, MyD88 and/or Mal transgenes. We further implicate TLR4 in LPS-induced HBD2 production by demonstrating HBD2 expression in LPS non-responsive HEK293 cells transfected with a TLR4 expression plasmid.

**Conclusion:**

This data defines an additional role for TLR4 in the host defense in the lung.

## Introduction

The lung represents the largest epithelial surface in the body and is a major portal of entry for pathogenic microorganisms. It employs a number of efficient defense mechanisms to eliminate airborne pathogens encountered in breathing, including the specific innate and adaptive immune responses, which represent a dynamic interaction of host and pathogen. Lipopolysaccharide (LPS) is an important antigenic component of Gram-negative bacteria, and is a potent stimulus to local and systemic immune responses. The human receptor for LPS is Toll-like-receptor 4 (TLR4) [1].

TLRs are a family of pattern recognition receptors whose pivotal importance in orchestrating the innate immune response is widely accepted. Binding of ligand activates a signaling cascade involving TRAF6, IKKs and I-κBs, culminating in NF-κB translocation to the nucleus [1]. NF-κB regulates the inducible expression of cytokines, chemokines, adhesion molecules and acute phase proteins which activate cellular immune responses [2]. TLR signaling pathways arise from intracytoplasmic Toll/IL-1 receptor (TIR) domains, which are conserved among TLRs and TIR domain-containing adaptor proteins such as MyD88, Mal/TIRAP and TRIF/TICAM-1. These adaptor proteins confer specificity on TLR signaling, with Mal specifically involved in MyD88-dependent signaling via TLR2 and TLR4, and TRIF in the MyD88-independent TLR3- and TLR4- signaling [3]

The mammalian innate immune system produces a variety of anti-microbial peptides (AMPs) as part of its host defense repertoire. The defensins are a broadly dispersed group of AMPs, and are classified according to their molecular structure into three distinct families: the α-, β- and the θ-defensins. Unlike α-defensins, which are produced mainly by neutrophils, β-defensins are produced directly by epithelial cells, and combat infection both through direct microbicidal action and by modulation of cell-mediated immunity [4-7]. To date, four human β-defensins (HBD) have been identified (HBD1-4), although genomic studies suggest more have yet to be discovered [8,9]. In contrast to HBD1, which is constitutively and stably expressed, HBD2 expression is induced in response to infective stimuli, including Gram-negative and, less potently, Gram-positive bacteria or their components or to proinflammatory stimuli including tumor necrosis factor α (TNFα) and interleukin-1β (IL-1β) *in vitro *[10,11].

Like other defensins, HBD2 has a broad spectrum of antimicrobial activity, displaying potent microbicidal activity against many Gram-negative bacteria and less potent bacteriostatic activity against Gram-positive bacteria [11]. It has recently been demonstrated that activation of TLR2 by bacterial lipoprotein results in up regulation of HBD2 in tracheobronchial epithelium [12]. LPS and Gram-negative bacteria such as mucoid *P. aeruginosa *are a more potent stimulus for HBD2 production, which in turn has anti-bacterial activity predominantly against Gram-negative bacteria. Colonisation and infection due to Gram-negative bacteria are important in many pulmonary diseases including severe COPD [13] and Cystic Fibrosis [14]. Production of HBD2 by respiratory epithelium is an important component of host defense against Gram-negative organisms, and understanding of the signaling pathways involved may further our understanding of and guide future therapeutic strategies in these diseases.

Cultured intestinal epithelial cells have been shown to produce HBD2 in response to LPS following transfection with TLR4 and MD2 [15]. Although CD-14 is known to be critical to LPS-induced HBD2 production in airway epithelium [16], the role of TLR4 in transcriptional regulation of HBD2 in respiratory epithelium has not been established. Indeed, the importance of the respiratory epithelium in the innate immune response to LPS has been called into question by some recent publications [17,18]. In this study we demonstrate TLR4 expression in A549 pulmonary epithelial cells and production of HBD2 in response to LPS. We examine the effect of modulation of TLR4 by receptor blockade and expression of a dominant negative TLR4 construct on induced expression of HBD2. We show that LPS-unresponsive HEK293 cells can produce HBD2 in response to LPS following transfection with TLR4 and MD2 transgenes and demonstrate that the adaptor proteins MyD88 and Mal are involved in transcriptional regulation of HBD2 in response to LPS.

## Methods

### Cell lines and culture

The human embryonic kidney cell line, HEK293, (ECACC-85120602) was obtained from the European Collection of Cell Cultures. Cells were cultured at 37°C in 5% CO_2 _in Eagle's minimal essential medium (EMEM, Biowhittaker) supplemented with 10% fetal calf serum (FCS), 1% L-glutamine, 1% penicillin/streptomycin, 1% NEAA (Gibco-BRL). The type II-like human lung epithelial cell line A549, (European Collection of Cell Cultures, Porton Down, UK) were cultured in Ham's F12 (Gibco-BRL), 10% FCS, 1% penicillin/streptomycin. Prior to agonist treatment, cells were washed with serum-free EMEM/F12 and placed under serum-free conditions or in serum containing 1% FCS for LPS stimulation experiments, including control conditions.

### Reverse Transcription (RT)-PCR

RNA isolation and cDNA synthesis were performed as previously described [19]. The integrity of RNA extraction and cDNA synthesis was verified by PCR by measuring the amounts of GAPDH cDNA in each sample using GAPDH-specific primers to generate a 211 bp product. PCR reactions were performed using standard conditions [19] with 50 pmol each of gene-specific primers (Table 1). After an initial step of 95°C for 5 min, thermocycling conditions were 35 cycles of 95°C for 30 sec, 55°C (TLR4 or CD14) or 58°C (MD2, Mal and GAPDH) for 30 sec and 72°C for 1 min per kb, followed by a final extension step of 72°C for 10 min. The more abundant GAPDH was amplified using 25 cycles. Control PCR reactions using an RNA template failed to generate any products. Products were resolved on 1.5% TBE agarose gels containing 0.5 μg/ml ethidium bromide (Sigma) and images were captured using the GeneGenius Gel Documentation and Analysis System (Syngene), analysed by densitometry and compared in a semi quantitative manner using ImageMaster^® ^TotalLab Software (Amersham Pharmacia, Amersham, UK). The ratio of PCR fragment intensities of HBD2 relative to GAPDH was determined. All expression values were verified by at least two independent RT-PCRs.

**Table 1:** 

**Gene (Accession No.)**	**Primers (5'-3')**	**Bases**	**Product Size**
TLR4 (NM_003266)			
F	AGATGGGGCATATCAGAGC	569–587	481 bp^a^
R	GTCCATCGTTTGGTTCTGG	1068–1050	
CD14 (NM_000877)			
F	TACTCCCGCCTCAAGGAA	459–476	197 bp
R	GCTTGGGCAATGCTCAGT	655–638	
MD2 (NM_000877)			
F	GCAACTCATCCGATGCA	95–112	225 bp
R	CATCAGATCCTCGGCAAA	319–302	
HBD2 (NM_AF071216)			
F	GGTATAGGCGATCCTGTTACC TGC	2688–2709	202 bp
R	TCATGGCTTTTTGCAGCA TTTTGTTC	4542–4567	
GAPDH (BC004109)			
F	AACTCTGGTAAAGTGGAT	122–138	211 bp
R	TACTCAGCGCCAGCATCG	333–316	

^a ^bp, base pairs

### IL-8 Production

Cells (1 × 10^5^) were left untreated or stimulated with LPS from Pseudomonas aeruginosa 01, human recombinant TNF-α or IL-1β (R&D systems). IL-8 protein concentrations in the cell supernatants were determined by sandwich ELISA (R & D Systems, U.K.). All assays were performed in duplicate or triplicate a minimum of three times.

### Preparation of membrane and cytosolic protein extracts

Cells were suspended in 1 ml ice-cold PBS and pelleted by centrifugation at 10,000 rpm for 5 min at 4°C. Supernatant was removed and the pellet resuspended in 100 μl hypotonic buffer A (5 mM Tris (pH 6.8), 2 mM EDTA, leupeptin 5 μg/ml, pepstatin 0.7 μg/ml, benzamidine 5 μg/ml, PMSF 1 mM)(Sigma, Ireland). Cellular components were separated by ultracentrifugation at 55000 rpm × 20 min at 4°C. Supernatant, which constituted cytosolic and nuclear fractions, was removed and stored at -20°C. The pellet consisting of the membrane fraction was resuspended in hypotonic buffer B (20 mM Tris HCl pH 6.8%, 150 mM NaCl, 10 mM EDTA, 1 mM EGTA, 1% Triton X100, leupeptin 5 μg/ml, pepstatin 0.7 μg/ml, benzamidine 5 μg/ml, PMSF 1 mM) by forcing the pellet through a 22 G needle 5–8 times. Protein concentrations of extracts were determined by the method of Bradford, and stored at -20°C.

### Western Blot analysis

Extracts (10 μg of protein) were separated by electrophoresis on 10% SDS-polyacrylamide gels and transferred to nitrocellulose, blocked with 0.2% I-Block (Tropix, MA) and PBS containing 0.1% Tween-20 (Sigma, Ireland). TLR4 protein was detected using rabbit anti-TLR4 (sc-10741 Santa Cruz Biotechnology, diluted 1:200), horse-radish peroxidase-conjugated anti-rabbit IgG (Tropix, MA) and chemiluminescent LumiGlo Reagent A and Peroxide Reagent B (New England Biolabs) according to the manufacturer's instructions.

### Laser Scanning Cytometry

For analysis by laser cytometry, 5 × 10^4^cells/well were seeded in 8 well chamber slides (Nunc). Slides were fixed in methanol (AnalaR) for 5 minutes and labelled with goat anti-HBD-2 antibody (a gift from Dr Paul McCray, University of Iowa, Iowa City, IA, USA) 1:100 dilution) or isotype control (1:1000 dilution of 1 mg/ml stock of isotype goat IgG) and incubated at 4°C in the dark for 30 min. Slides were washed three times in PBS and probed with 1:10 dilution of anti-goat IgG fluorescein isothiocyanate (FITC, Dako, Glostrup, Denmark) at 4°C in the dark for 30 min. The washing step was repeated and cells were permeabilised in a 1:1 ratio of permeabilisation solution (Dako) and 0.2 μg/ml solution of propidium iodide (PI, Molecular Probes, Leiden, The Netherlands). Slides were washed in PBS and HBD-2 expression was quantified on a CompuCyte laser scanning cytometer (CompuCyte, Cambridge, MA, USA). Cellular fluorescence of at least 5 × 10^3 ^cells was measured by laser scanning cytometry. Fluorescence excitation was provided by a 488 nm laser line. Orange (PE) and green (FITC) fluorescence were measured at 588 +/- 10 nm or 530 +/- 20 nm, respectively. The threshold contour was set on scatter or orange to detect all cells as appropriate. Artificially contoured debris was gated out based on contour size. Aggregated cells were gated out using an algorithm in the LSC software that finds and marks multiple cells. Individual TLR4 or HBD-2-expressing cells were identified and quantified using CompuCyte software on the basis of integrated green fluorescence reflecting binding of anti-goat FITC antibody.

### Transfection and reporter gene studies

Cells (1.5 × 10^5^) were transfected with plasmid DNA (dominant negative (Δ) Mal (Mal P/H), ΔMyD88, ΔTLR4, MD2, wild type TLR4 or CD4-Toll plasmid) and/or 1 μg HBD2 promoter-linked luciferase reporter plasmid [20] using TransFast (Promega) according to the manufacturer's instructions. ΔMyD88 contains only a functional TIR domain and lacks the death domain required for downstream signaling, while Mal P/H is a dominant negative version of Mal with a proline to histidine point mutation in box 2 of the TIR domain. ΔTLR4 lacks an intracytoplasmic signaling domain and CD4-Toll is a constitutively active chimera of the extracellular domain of CD4 fused to the transmembrane and cytosolic domains of TLR4 [21]. Uniform transfection efficiencies were achieved by measuring expression from co-transfected luciferase or β-galactosidase expression plasmids, as appropriate. initially optimizing transfection conditions using a constitutive luciferase expression vector, pGL3-control (Promega). In all experiments, equal amounts of empty vector plasmid DNA was used in control cells, such that the total amount of transfected DNA remained constant. After 48 h cells were lysed with Reporter Lysis Buffer (1×) (Promega) (300 μl/well), protein concentrations were determined, and reporter gene activity was quantified by luminometry (Wallac Victor^2^, 1420 multilabel counter, Turku, Finland) using the Promega luciferase assay system according to the manufacturer's instructions.

### Statistical analysis

Data were analyzed with GraphPad Prism 3.0 software package (GraphPad Software, San Diego, CA). Results are expressed as mean ± S.E. and were compared by Mann-Whitney test. Differences were considered significant when the *P *value was ≤ 0.05.

## Results

### A549 cells respond to LPS with production of IL-8 and up regulation of HBD2

**Figure 1:**
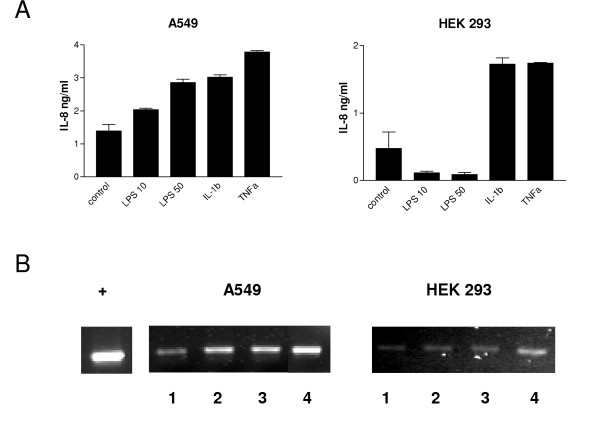
LPS-induced IL-8 and HBD2 expression in A549 and HEK293 cells. (A) A549 or HEK293 cells (3 × 10^5^/ml) were left untreated (control) or stimulated for 24 h with LPS (10 or 50 μg/ml), IL-1β (100 ng/ml) or TNFα (10 ng/ml). Levels of IL-8 in supernatants were measured by ELISA and values are expressed as ng/ml. Assays were performed in duplicate a minimum of three times. Values are expressed as mean+/- S.E. (n = 3). (B) Total RNA was extracted from A549 or HEK293 cells, reverse transcribed into cDNA and used as a template in PCR reactions using HBD2 gene-specific primers. Products were electrophoresed in 1.5% TBE agarose gels containing 0.5 μg/ml ethidium bromide and visualized under UV. + represents positive control PCR reaction and lanes 1–4 represent untreated cells and cells stimulated for 24 hours with LPS (10 μg/ml), IL-1β (100 ng/ml) or TNFα (10 ng/ml), respectively. Gels are representative of three independent experiments using cultures from different time points.

Signaling via TLR4 by LPS activates NF-κB and upregulates a variety of pro-inflammatory genes, including IL-8. We investigated LPS responsiveness in A549 and HEK293 cells using IL-8 protein production as a surrogate of LPS responsiveness. Figure 1A shows that A549 cells dose-dependently induced IL-8 protein expression in response to stimulation with LPS to levels similar to those induced by IL-1β. In contrast HEK293 cells failed to induce IL-8 expression in response to LPS, but did respond to IL-1β or TNFα stimulation with increased IL-8 expression.

We next investigated the effect of LPS stimulation on HBD2 gene expression in both cells lines. Compared to untreated cells, LPS induced HBD2 expression at both 10 and 50 μg/ml in A549 cells (Figure 1B). Densitometric analysis quantified these increases to be 2- and 3.6-fold, respectively. IL-1β was used as a positive control and increased HBD2 expression over 30-fold. There was no response to LPS in the HEK293 cells however both IL-1β and TNFα did up regulate HBD2 by a factor of 1.8 and 1.5 respectively.

### Airway epithelial cells express TLR4 on the cell surface

**Figure 2:**
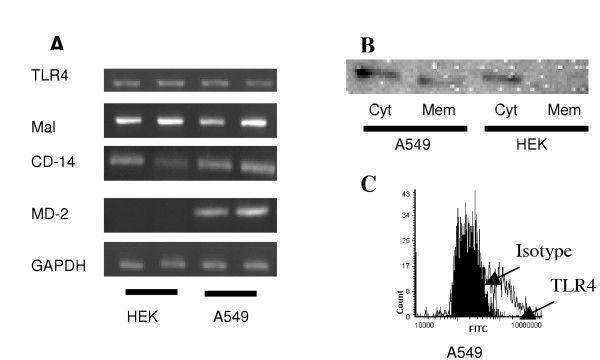
Characterization of A549 and HEK293 cell lines. (A) Duplicate samples of total RNA was extracted from 1 × 10^6 ^HEK293 (HEK) and A549 cells, reverse transcribed into cDNA and used as a template in PCR reactions using TLR4, Mal, CD14, MD2 and GAPDH gene-specific primers. Products were electrophoresed in 1.5% TBE agarose gels containing 0.5 μg/ml ethidium bromide and visualized under UV. (B) Western blot analysis of membrane (mem) and cytosolic (cyt) extracts (10 μg) from A549 and HEK293 cells probed with an anti-TLR4 antibody. Data are representative of three separate experiments. (C) For fluorescence microscopy, A549 cells (2 × 104) were grown in chamber slides, Fc-blocked and labelled with anti-TLR4 (clear) or isotype control antibodies (solid) and fluorophore-conjugated detection antibodies. TLR4 expression was quantified by laser scanning cytometry.

We characterized the HEK293 cell line to determine whether it lacked a critical factor for LPS responsiveness. RTPCR revealed that the HEK293 cells, similar to the A549 cells, express TLR4, Mal, and CD14 mRNA but unlike the A549 cells do not express MD2 mRNA (Figure 2A). MD2 is a secreted protein who's interaction with LPS and CD-14 is necessary for the cellular response to LPS [22]

We performed western immunoblotting of cytosolic and membrane fractions from HEK293 and A549 cells to detect TLR4 protein expression. Figure 2B shows that TLR4 was present in both fractions from the A549 cells but was not evident in membrane fractions isolated from HEK293 cells. Next we quantified cell surface expression of TLR4 on A549 by fluorescence microscopy. In accord with the findings of Monick et al [17] but in contrast to Guillot, [23] we detected TLR4 on the surface of A549 cells (Figure 2C).

### LPS-induced HBD2 gene and protein expression in A549 cells requires TLR4

**Figure 3:**
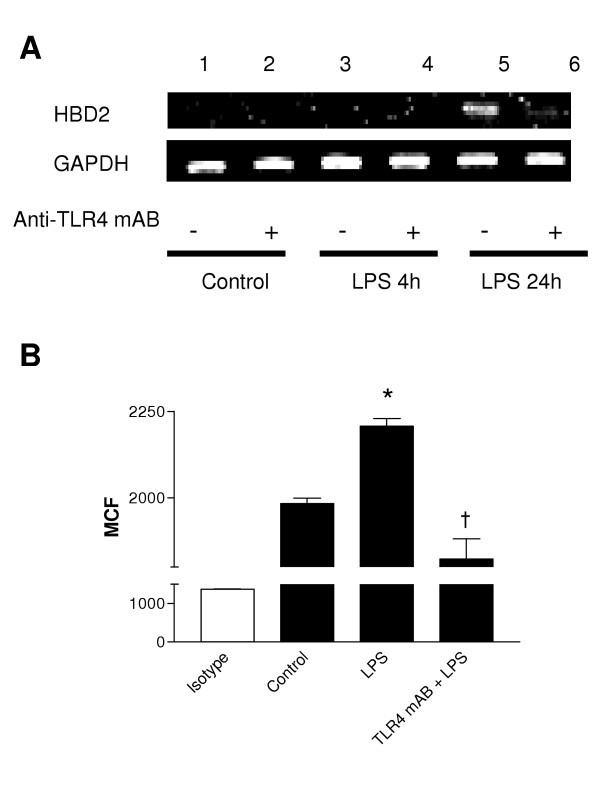
LPS-induced HBD2 expression in A549 cells requires TLR4. A459 cells were incubated with an isotype control or anti-TLR4 neutralizing antibody (Anti-TLR4 mAB 5 μg/ml, 30 min) then, (A) left untreated or stimulated with LPS (10 μg) for 4 or 24 hours. Total RNA was extracted, reverse transcribed into cDNA and used as a template in PCR reactions using HBD2 gene-specific primers. Products were electrophoresed in 1.5% TBE agarose gels containing 0.5 μg/ml ethidium bromide and visualized under UV. Gels are representative of three independent experiments or (B) left untreated or stimulated with LPS (10 μg) for 24 hours, Fc-blocked and labeled with anti-HBD2 (solid) or isotype control antibodies (clear) and fluorophore-conjugated detection antibodies. HBD2 expression was quantified by laser scanning cytometry, as described, and data from three experiments is presented. HBD2 expression is expressed as Mean Channel Fluorescence (MCF) + SEM. (* P < 0.05 vs control, † P < 0.05 vs control + LPS).

Next the role of TLR4 in LPS-induced regulation of HBD2 (Figure 3A) in A549 cells was investigated. Compared to untreated cells (lane 1), LPS increased HBD2 expression at 24 h (lane 5). This effect was blocked by pre-treatment with a TLR4 neutralizing antibody (eBioscience, Clone HTA125, Cat. No. 16–9917) (lane 6). An isotype control antibody had no effect (data not shown). A similar effect was demonstrated at protein level using laser scanning cytometry. Stimulation with LPS resulted in a small but statistically significant increase in HBD2 protein above control (P < 0.03). This effect is inhibited by pretreatment with the TLR4 blocking antibody (P < 0.03).

### Dominant negative TLR4 inhibits LPS-induced HBD2 expression in A549 cells

**Figure 4:**
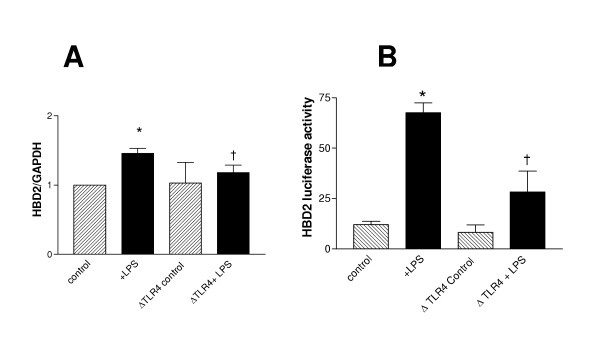
ΔTLR4 inhibits LPS-induced HBD2 expression in A549 cells. A549 cells (1.5 × 10^5^) were transfected with pcDNA3 (empty vector) or a ΔTLR4 expression plasmid. 24 h post transfection, cells were left untreated or stimulated with LPS (10 μg/ml) for 24 h. (A) Total RNA was extracted, reverse transcribed into cDNA and used as a template in semi-quantitative PCR reactions using HBD2 and GAPDH gene-specific primers. HBD2 expression was given an arbitrary value of 1 in control cells. Data are expressed as mean +/- S.E. and are obtained from three experiments (* *P *< 0.05 vs control, † P < 0.05 vs control + LPS). (B) Duplicate experiments were performed in cells cotransfected with a HBD2 promoter-linked luciferase reporter plasmid. Cells were lysed and reporter gene activity was quantified by luminometry. Data are expressed as HBD2 luciferase activity (n = 3). (* *P *< 0.05 vs control, † P < 0.05 vs control + LPS)

In order to further demonstrate the functional role of TLR4 in LPS-induced HBD2 expression, we examined the effect of functionally ablating TLR4 by over expression of a functionally inactive ΔTLR4 construct in A549 cells. Because the upregulation of HBD2 by LPS as measured by semi-quantitative RTPCR was small, albeit statistically significant, we further quantified the effect by luciferase activity of a co-transfected HBD2 promoter-linked luciferase construct (Figure 4). LPS increased both HBD2 mRNA expression (Figure 4A) (P ≤ 0.05) and HBD2 promoter activity (Figure 4B) (P ≤ 0.03) however over expression of ΔTLR4 significantly inhibited both effects (P ≤ 0.05 and 0.03 compared to LPS-treated empty vector-transfected cells, respectively).

### TLR4/MD2 transgene expression confers LPS-responsiveness on HEK293 cells

**Figure 5:**
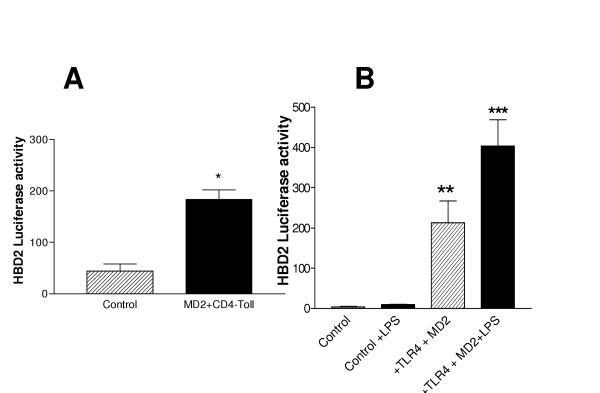
TLR4/MD2 transgene expression confers LPS-responsiveness on HEK293 cells. HEK293 cells (1.5 × 10^5^) were cotransfected with MD-2 and CD4/Toll or full-length TLR4 expression plasmids and a HBD2 promoter-linked luciferase reporter gene. Equal amounts of the corresponding empty vector were transfected into control cells such that the total amount of transfected DNA remained constant. Uniform transfection efficiencies were confirmed using a β-galactosidase reporter plasmid. 24 hours post transfection, cells were left untreated or stimulated with LPS (10 μg/ml) then lysed and reporter gene activity was quantified by luminometry. Data are expressed as HBD2 luciferase activity. Assays were performed in duplicate and are representative of at least three separate experiments. (* P < 0.05, ** P < 0.005, *** P < 0.001 vs control).

Having established the inhibitory effect of neutralizing TLR4 in A549 cells by receptor blockade and over-expression of a dominant negative construct, we next determined whether HEK293 cells could be rendered responsive to LPS by expression of functional TLR4 and MD2. Expression of CD4/Toll (a constitutively active TLR4 chimera [21]) and MD2 resulted in significant induction of the HBD2 promoter (Figure 5A).

Stimulation of HEK293 cells with LPS has no effect on HBD2 promoter activity (Figure 5B). However transfection with MD2 and TLR4 transgenes resulted in significant up regulation of HBD2 promoter activity (P ≤ 0.005 compared to LPS-treated empty vector-transfected cells), an effect that was further significantly augmented by stimulation with LPS (P ≤ 0.05 compared to untreated MD2/TLR4-transfected cells).

### Expression of dominant negative MyD88 and Mal constructs inhibits LPS-stimulated HBD2 expression

**Figure 6:**
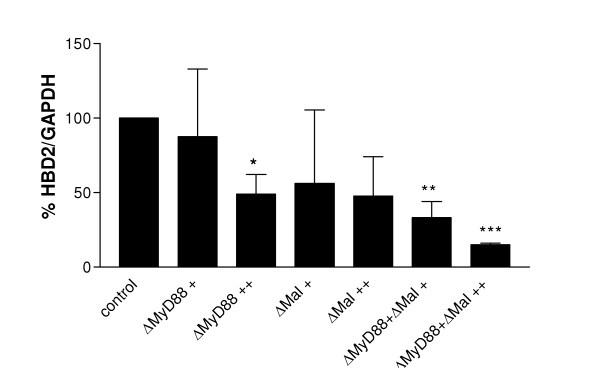
ΔMyD88 and ΔMal inhibit LPS-induced HBD2 expression in A549 cells. A549 cells (1.5 × 10^5^) were transfected with pCDNA3.1 or pDC304 (empty vectors), ΔMyD88 or Mal P/H expression plasmids as indicated. 24 h post transfection, cells were stimulated with LPS (10 μg/ml) for 24 h. HBD2 expression was measured by semi-quantitative RTPCR. Expression in LPS-treated cells was ascribed a value of 100 %. Data shown are mean+/- S.E. (n = 3). Regarding amounts of transfected DNA, ΔMyD88 + and ++ represent 100 and 200 ng of dominant negative MyD88 plasmid DNA respectively while Mal + and ++ represent 50 and 100 ng of dominant negative Mal plasmid DNA respectively. (* P < 0.05, ** P < 0.01, *** P < 0.005 vs control + LPS)

Having demonstrated that LPS up regulates HBD2 expression via TLR4, we went on to elucidate the pathway by which the signal is transduced to the nucleus. Figure 6 shows that transfection of dominant negative constructs of the TIR domain containing adaptor proteins MyD88 and Mal alone or in combination dose-dependently inhibited HBD2 expression in response to LPS. Technical restrictions of performing transfection experiments in chamber slides precluded measurement of HBD2 protein in this part of the study. We would intuitively expect a similar qualitative effect in HBD2 mRNA and protein expression, as demonstrated in Figure 3.

## Discussion

Induced expression of HBD2 in response to infective and pro-inflammatory stimuli represents an immediate and dynamic response by the host epithelium to potential infection, and the mechanism by which this occurs has been the subject of much recent investigation. Here we provide evidence for a critical role for TLR4 in LPS-induced HBD2 expression in airway epithelial cells. The data show that A549 cells respond to LPS with increased HBD2 gene and protein expression and that these effects can be blocked by a TLR4 neutralizing antibody or transfection with functionally inactive TLR4, MyD88 or Mal transgenes. We further implicate TLR4 in LPS-induced HBD2 expression by demonstrating that expression of functional TLR4 and MD2 (a co-factor which is unique to and necessary for TLR4 activity) in HEK293 cells confers LPS responsiveness to these cells, which can lead to HBD2 induction.

There is some discrepancy in the literature regarding surface expression of TLR4 in A549 cells. Guillot and colleagues reported that TLR4 is not expressed on the surface of A549 cells, but is compartmentalized to the intracellular compartment [23], while Monick et al. demonstrated low level surface expression on the same cells [17]. Our data showed low-level surface expression of TLR4 by LSC, and TLR4 was also detectable in membrane fractions by Western blotting. Further evidence of surface expression comes from the ability to block the receptor with anti-TLR4 monoclonal antibody. Surface expression of TLR4 has been demonstrated on other respiratory epithelial cells including human bronchial epithelial cells [24] and human airway cells in primary culture, where it was found in a more basolateral distribution. The findings of higher levels of TLR4 in the cytoplasmic fraction is also relevant, as internalisation of LPS in A549 cells has been reported as early as 4 hours after LPS challenge [25], and this internalisation modulated expression of ICAM-1 and TNF. It remains unclear whether LPS-TLR4 co-localisation intracellularly activates the same signalling cascade as at the cell membrane.

Similar controversy exists regarding the LPS responsiveness of A549 cells. Previous studies have suggested that A549 cells were hyporesponsive to LPS, at doses of up to 100 μg/ml [26-28]. This was not the case in our study. A549 cells responded to 10 μg/ml LPS with significant up regulation of both HBD2 and IL-8. One potential reason for this difference is the type of LPS used. Previous studies used *E. coli *LPS. *P. aeruginosa *is an important respiratory pathogen, particularly in patients with lung diseases such as cystic fibrosis (CF) [14]. *E. coli*, in contrast, is more important in the gastrointestinal and genitourinary tract, and as an important cause of septic shock [29]. Mucoid strains of *Pseudomonas *have been demonstrated to induce HBD2 in respiratory epithelia including A549 cells [10]. For these reasons, we used *Pseudomonas *LPS in our study. CD-14 is a glycoprotein which, together with TLR4 and LPS Binding Protein (LBP), forms the LPS signaling complex, and exists in membrane bound and soluble (in serum) forms. Soluble CD14 is required for LPS signaling in A549 cells [30], and it is therefore important that LPS stimulation is performed in the presence of serum, as in our study.

Different types of LPS differ in their ability to stimulate cells. Structural differences in LPS, most commonly in the *O*-polysaccharide chain [31], may result in different biological properties. *E. coli *LPS is highly toxic in its ability to propagate the systemic inflammatory response syndrome, principally through activation of monocytes. *P. aeruginosa *LPS differs from *E. coli *LPS both in the *O*-polysaccharide side chain and in the Lipid A component, and stimulates significantly less endotoxic effect [32], but induces sustained airway inflammation in a number of chronic lung diseases including CF and diffuse panbronchiolitis [33]. *Pseudomonas *LPS has been shown to be significantly more potent than LPS from a number of different strains of *E. coli *in its ability to stimulate IL-8 and granulocyte colony-stimulating factor (G-CSF) from respiratory epithelial cells, including A549 cells [34]. The reason for this is not clear, but structural differences may determine its processing by the TLR4/MD2 complex, either directly or through its interaction with the host plasma membrane [35,36]. Basolateral expression of TLR4 has been reported in pulmonary epithelial cells [37], and this is particularly interesting given that *Pseudomonas *elastase has been shown to increase epithelial permeability by its effect on tight junctions [38], thereby potentially increasing access of *Pseudomonas *LPS to the basally expressed TLR4.

Previous work by Becker et al [16] in primary human tracheobroncial cells shows a clear increase in HBD2 protein in response to LPS by western blotting, although another recent study in primary airway epithelial cells reports low expression of MD2 limiting LPS responsiveness [18]. MD2 was induced in response to pro-inflammatory cytokines and bacterial products [18], while MD2 expression in A549 cells is enhanced along with TLR4 following infection with RSV [17]. While differences speak to the limitations of using a cultured cell model, as well as to the variable responses of cultured primary cells, they also reinforce the critical importance of both TLR4 and MD2 in the signalling pathway. A549 cells clearly expressed MD2 and TLR4 and responded to Pseudomonas LPS in our hands. LPS stimulation resulted in a 5-fold increase in HBD2 promoter linked activity. The increase in HBD2 protein, though statistically significant, was small in absolute terms raising questions regarding the physiological significance of LPS-induced epithelial derived HBD2 production.

Indirect activation of epithelial cells by proinflammatory cytokines released by stimulated alveolar macrophages may be more important at lower concentrations of LPS [26], while direct stimulation of the epithelial cells may become important when bacterial load is high, where TLR4 and MD2 expression is enhanced, or following internalisation of LPS. The kinetics of the epithelial response in this and other studies [16,20] may also be relevant, with direct stimulation of the epithelial cells providing a slower but potentially more sustained release of HBD2 than that induced by inflammatory mediators. Indeed, recent studies of cellular cross-talk between epithelial cells and mononuclear cells suggests co-localisation of these cells may result in a more pronounced immune response *in vivo *[25]. Further work using primary cell culture and co-culture with immune cells mimicking physiologic conditions is required to clarify the relative contributions of these cells to HBD2 production *in vivo*.

The mechanism by which LPS induces HBD2 expression in respiratory epithelium has not been previously reported. Indirect evidence for involvement of a TLR came first from Diamond and colleagues [39] in 1996, who described CD-14 dependent LPS induced production of an anti-microbial peptide in bovine tracheal epithelium, and later from Becker and colleagues, who showed that HBD2 up regulation in response to LPS in human tracheobronchial cells was CD14 dependent (33). The GPI-linked CD14 receptor lacks a cytosolic domain and must interact with another receptor to transduce its signal to the nucleus [40]. Transcriptional regulation of HBD2 in response to LPS has also been shown to involve NF-κB [41], but the signaling pathway upstream of NF-κB has not been elucidated. Until recently, TLRs appeared to share a common signaling pathway downstream of their TIR domain. It is now known that individual TLRs utilize different adaptor proteins for signaling, thus conferring biological specificity in their response to activation by individual ligands [42,43]. MyD88 is involved in signaling from all TLRs with the exception of TLR3, whilst Mal is known to have a role in TLR2 and TLR4 intracellular signaling. Our data implicate both MyD88 and Mal in LPS-induced HBD2 expression and clearly demonstrates the critical role of TLR4 in LPS induction of HBD2, thus defining another role for TLR4 in pulmonary host defense. Along with the previously well-defined functions of TLR4 in induction of a large number of cytokines, chemokines and adhesion molecules that activate phagocytosis and the adaptive immune responses, activation of TLR4 in the epithelium results in a direct microbicidal response via production of a potent anti-microbial peptide.

Modulation of TLR4 expression in respiratory epithelium may critically affect the production of HBD2. Risk factors for respiratory tract infections include increased age and smoking [44], both of which may affect TLR4 expression. TLR4 expression in macrophages is reduced in aged mice, who have a blunted cytokine response to LPS [45]. Cigarette smoke has also been demonstrated to reduce LPS responsiveness in alveolar macrophages [46]. As LPS is an active component of cigarette smoke [47], and down regulation of TLR4 expression by LPS in cigarette smoke, otherwise known as LPS tolerance, may result in impaired HBD2 production in response to Gram-negative pathogens, facilitating colonization and infection. This is the subject of ongoing work in our laboratory. Similarly, therapies for septic shock aimed at inhibiting LPS signaling by blockade of the TLR4 receptor [48] may result in increased susceptibility to nosocomial Gram-negative pneumonia through impaired HBD2 induction.

HBD2 is an important component of host defense in the lung. This study defines a critical role for TLR4 in induced expression of HBD2 in response to LPS and highlights the potential effect of modulation of TLR4 and the accessory proteins MyD88 and Mal expression on pulmonary production of this potent anti-microbial peptide. We also provide important information regarding the cellular responses of A549 cells, a cultured cell model which is widely used in studies of host defense.
